# From fruit growth to ripening in plantain: a careful balance between carbohydrate synthesis and breakdown

**DOI:** 10.1093/jxb/erac187

**Published:** 2022-05-05

**Authors:** Nadia A Campos, Sophie Colombié, Annick Moing, Cedric Cassan, Delphine Amah, Rony Swennen, Yves Gibon, Sebastien C Carpentier

**Affiliations:** Biosystems Department, KULeuven, 3001 Leuven, Belgium; INRAE, Fruit Biology and Pathology, Université De Bordeaux, UMR 1332, 33140 Villenave d’Ornon, France; INRAE, Fruit Biology and Pathology, Université De Bordeaux, UMR 1332, 33140 Villenave d’Ornon, France; INRAE, Fruit Biology and Pathology, Université De Bordeaux, UMR 1332, 33140 Villenave d’Ornon, France; IITA, Crop Breeding, Ibadan 200001, Oyo State, Nigeria; Biosystems Department, KULeuven, 3001 Leuven, Belgium; IITA, Crop Breeding, PO Box 7878, Kampala, Uganda; INRAE, Fruit Biology and Pathology, Université De Bordeaux, UMR 1332, 33140 Villenave d’Ornon, France; Biosystems Department, KULeuven, 3001 Leuven, Belgium; Bioversity International, Biodiversity for Food and Agriculture, 3001 Leuven, Belgium; SYBIOMA, KULeuven, 3001 Leuven, Belgium; University of California, Davis, USA

**Keywords:** Banana, fruit ripening, metabolite flux analysis, *Musa* species, proteomics, starch

## Abstract

In this study, we aimed to investigate for the first time different fruit development stages in plantain banana in order gain insights into the order of appearance and dominance of specific enzymes and fluxes. We examined fruit development in two plantain banana cultivars during the period between 2–12 weeks after bunch emergence using high-throughput proteomics, quantification of major metabolites, and analyses of metabolic fluxes. Starch synthesis and breakdown are processes that take place simultaneously. During the first 10 weeks fruits accumulated up to 48% of their dry weight as starch, and glucose 6-phosphate and fructose were important precursors. We found a unique amyloplast transporter and hypothesize that it facilitates the import of fructose. We identified an invertase originating from the *Musa balbisiana* genome that would enable carbon flow back to growth and starch synthesis and maintain a high starch content even during ripening. Enzymes associated with the initiation of ripening were involved in ethylene and auxin metabolism, starch breakdown, pulp softening, and ascorbate biosynthesis. The initiation of ripening was cultivar specific, with faster initiation being particularly linked to the 1-aminocyclopropane-1-carboxylate oxidase and 4-alpha glucanotransferase disproportionating enzymes. Information of this kind is fundamental to determining the optimal time for picking the fruit in order to reduce post-harvest losses, and has potential applications for breeding to improve fruit quality.

## Teaser

Combined analyses of the proteomes and metabolomes of plantain banana cultivars indicate the order of appearance and dominance of specific enzymes/fluxes during fruit development.

## Introduction

Fruit development is a complex phenomenon that encompasses several overlapping stages: cell division and enlargement, maturation, ripening, and senescence (Paul *et al.*, 2012). In general, fruits can be divided into two groups with contrasting ripening mechanisms, namely climacteric and non-climacteric. Climacteric fruits include tomato, avocado, apple, and banana, and are characterised by a strong production of ethylene and an increase in respiration during ripening (White, 2002). During maturation, two systems of ethylene are operational. In early maturation, system 1 is active, during which the rate of ethylene production is basal and there is an auto-inhibition of further production. As maturation progresses, this inhibition process is stopped and there is an auto-induction of ethylene production leading to the onset of ripening (system 2; Paul *et al.*, 2012). The most notable events occurring during fruit ripening are changes in colour, fruit softening, and starch breakdown/sweetening. In banana, these processes are highly associated with different cultivars (Cordenunsi-Lysenko *et al.*, 2019).

Edible bananas (*Musa* species) are parthenocarpic and hence the ovaries develop into seedless fruits without a pollination stimulus and hence without fecundation. The pulp-initiating cells are situated within the inner epidermis of the fruit pericarp and septa. In parthenocarpic bananas, these cells start to proliferate very rapidly after flowering (bunch emergence) (Ram *et al.*, 1962). The increase in cell number in the initiating region of the pulp continues up to about 4 weeks after bunch emergence (WAE), after which it declines and growth is then largely realized by cell enlargement (Ram *et al.*, 1962). Sugar deposition and starch synthesis in the pulp cells commence very early, and they become well established by 8 WAE. The first signs of disappearance of starch have been reported to be around 12 WAE (Ram *et al.*, 1962); however, this is dependent on the environment and on the cultivar. Depending on the cultivar, banana fruit have been reported to reach a starch content of between 12–35% of total dry weight during the period 4–8 WAE, after which the content drops to between 0–15% in the late stages of maturation (Soares *et al.*, 2011; Cordenunsi-Lysenko *et al.*, 2019). Plantains are part of a group of bananas that accumulate a large amount of starch. When they are ripe, plantains still have a high starch content, which affects their taste (Soares *et al.*, 2011). Hence, in contrast to sweet dessert bananas, plantains are consumed as a starch source and they are an important staple food in tropical and subtropical countries, being of special importance in West Africa (Vuylsteke *et al.*, 1993). The current practice is to harvest them when the fruits of the first hand show signs of ripening (Dadzie and Orchard, 1997). There are four main groups of plantain cultivars based on their inflorescence morphology: French, French Horn, Horn, and False Horn (Swennen and Vuylsteke, 1987; Swennen *et al.*, 1995). Genetically, the different cultivars are triploids and belong to the AAB genotype group. They are product of a natural cross between *M. acuminata* (A genotype) and *M. balbisiana* (B genotype) (Simmonds, 1962). Although the different plantain cultivars are morphologically quite diverse, genetically they are extremely uniform (Crouch *et al.*, 2000). The recent release of the B genome sequence suggested a dominance of genes related to starch metabolism, leading to higher starch accumulation during fruit development (Wang *et al.*, 2019).

Proteomics is a technology that has been successfully applied to study complex biological processes in fruit (Agrawal *et al.*, 2013; Bak *et al.*, 2013; Esteve *et al.*, 2013; Righetti *et al.*, 2015; Szymanski *et al.*, 2017; Campos *et al.*, 2018; Bhuiyan *et al.*, 2020), and its application to physiology and/or development in bananas has revealed important proteins that have given us a better understanding of these processes. However, most studies are focused on the Cavendish sweet banana, which is the most exported banana cultivar in the world (Agopian *et al.*, 2008; Toledo *et al.*, 2012; Asif *et al.*, 2014; Du *et al.*, 2016). In addition, very little information is available about early fruit development and starch formation, and the very first degradation steps. We have recently published the first proteome of detached fruit and a comparison of the proteomes of Cavendish and plantain during the final ripening process (Campos *et al.*, 2018; Bhuiyan *et al.*, 2020). Together with the recent update of the B genome sequence (Wang *et al.*, 2019), these studies have contributed to elucidating fruit development in plantain as well to determining the role of the B genome in fruit quality. In this current study, we aimed to investigate for the first time different fruit development stages and to gain insights into the order of appearance and dominance of specific enzymes/fluxes. We complemented proteome studies with analyses of biomass composition, which enabled the modelling of the metabolic fluxes in central carbon metabolism. The measurement of fluxes in fruits is extremely challenging, and hence they were predicted using constraint-based models based on a description of the metabolic network obtained through stoichiometric equations of reactions, and on the assumption of a pseudo-steady state and the choice of an objective function (Orth *et al.*, 2010). Such stoichiometric models describing central metabolism have already proved useful in tomato fruit to estimate fluxes throughout development (Colombié *et al.*, 2015, 2017). A better understanding of the mechanisms that regulate sugar primary metabolism during fruit development will be important for selecting hybrids with the best post-harvest traits. Plantain breeding efforts at the International Institute of Tropical Agriculture (IITA) at Ibadan, Nigeria, are essentially concentrated on a few cultivars of the French plantain type (Vuylsteke *et al.*, 1993). More knowledge about plantain physiology through proteomics and metabolic flux studies can improve such breeding programs and allow the use of other cultivars, including those most favoured by consumers.

## Materials and methods

### Biological material

All biological samples were collected from the IITA Experimental Field in Ibadan, Nigeria, during the period from October 2016 to February 2017.

Five banana plants of each of the Agbagba and Obino l’Ewai cultivars were selected and the same plants were followed throughout the experiment. One fruit per plant was collected from 2 weeks after bunch emergence (WAE) until the fruits reached full maturity. The collected fruits were cleaned and measurements of fruit length (L) and circumference (C) were taken. For the calculation of fruit volume our formula was based on (Simmonds, 1953). To determine the correlations between fruit weight, calculated fruit volume, and real fruit volume, the real volumes of representative fruits were measured by submerging them in water in a measuring cylinder. For the remainder of the fruits, the volume was calculated as: Volume (cm^3^) = [Fruit length × (Fruit circumference)^2^ × 0.0616] + 0.3537. After determination of fruit length and circumference, the fruits were separated from their peels, cut into pieces and stored at –80 °C until lyophilization. Samples were lyophilized to ensure safe transportation from the growing site in Nigeria to the laboratory in KU Leuven in Belgium, and to facilitate the protein and metabolite extraction process (Carpentier *et al.*, 2007).

### Protein extraction, quantification, identification, and annotation

Extractions were performed following the phenol-extraction/ammonium-acetate precipitation protocol described previously (Carpentier *et al.*, 2005; Buts *et al.*, 2014). Samples at 2 WAE and 4 WAE could not be analysed for proteomics due to the presence of many interfering compounds that affected the correct application of the protocol.

After extraction, 20 µg of proteins were digested with trypsin (Trypsin Protease, MS Grade) and purified using Pierce C18 Spin Columns (both ThermoFisher Scientific). The digested samples (0.5 µg per 5 µl) were separated in an Ultimate 3000 UPLC system and then in a Q Exactive Orbitrap mass spectrometer (both ThermoFisher Scientific) as described by van Wesemael *et al.* (2018). For protein quantification, we used the Progenesis^®^ software (Nonlinear Dynamics). In this software we used MASCOT v.2.2.06 (Matrix Science) against the Musa V2 database of *M. acuminata* and *M. balbisiana* (Martin *et al.*, 2016; Wang *et al.*, 2019) (157 832 proteins). Tandem mass spectra were extracted by Progenesis. All MS/MS spectra were searched with a fragment ion mass tolerance of 0.020 Da and a parent ion tolerance of 10.0ppm. Carbamidomethyl of cysteine was specified in Mascot as a fixed modification. Deamidation of asparagine and glutamine and oxidation of methionine were specified in Mascot as variable modifications and the results were reintroduced in Progenesis. Scaffold (version Scaffold_4.11.0, Proteome Software) was used to validate the MS/MS-based peptide and protein identifications. Mascot and X! Tandem were searched with the following specified variable modifications: Glu->pyro-Glu of the N-terminus, ammonia-loss of the N-terminus, gln->pyro-Glu of the N-terminus, deamidation of asparagine and glutamine, and oxidation of methionine. Peptide identifications were accepted if they could be established at >95% probability by the Peptide Prophet algorithm with Scaffold delta-mass correction (Keller *et al*., 2002; Searle, 2010). Protein identifications were accepted if they contained at least one identified peptide. Protein probabilities were assigned by the Protein Prophet algorithm (Nesvizhskii *et al.*, 2003). Proteins that contained similar peptides and could not be differentiated based on MS/MS analysis alone were grouped to satisfy the principles of parsimony. Proteins sharing significant peptide evidence were grouped into clusters. A protein false-discovery rate of 0.8% and a spectral false-discovery rate of 0.04% was observed by searching the reverse concatenated decoy database (157 832 proteins).

Gene annotations were taken from the banana Hub (Droc *et al.*, 2013) and verified in the Plant Metabolic Network (Hawkins *et al.*, 2021) and Prosite (https://www.expasy.org/resources/prosite). Subcellular predictions were analysed using the software DeepLoc 1.0 (Almagro Armenteros *et al.*, 2017).

### Metabolic analysis

To complement the proteomics data and gain further insights regarding plantain fruit development, we analysed major metabolic traits in pulp. The metabolite analyses were performed at the Bordeaux Metabolome facility. Metabolites were extracted from 10 mg aliquots of lyophilized ground samples via three extractions with ethanol-buffer mixtures successively composed of 80% ethanol, 80% ethanol, and 50% ethanol and 10 mM Hepes/KOH buffer (pH 6). The supernatants were collected and pooled for measurement of soluble metabolites. Glucose, fructose, and sucrose were measured enzymatically (Stitt *et al.*, 1989). Glucose 6-phosphate (G6P), fructose 6-phosphate (F6P), and glucose 1-phosphate (G1P) were measured using an enzyme cycling assay (Gibon *et al.*, 2002). Malate was measured enzymatically as described by Mollering (1985). Total free amino acids were measured using fluorescamine (Bantan-Polak *et al.*, 2001). Polyphenols were measured using Folin–Ciocalteu’s reagent (Blainski *et al.*, 2013). In order to quantify the total protein content, the pellets remaining after the extraction of the soluble metabolites were resuspended in 100 mM NaOH and then heated for 20 min. After centrifugation at 5000 *g* for 5 min, the total protein content was measured using Coomassie Blue (Bradford, 1976). After neutralization with HCl, starch was quantified in the pellets as described previously (Hendriks *et al.*, 2003). Finally, the pellet was washed twice with water and twice with 96% ethanol (v/v), dried, and weighed to estimate the cell wall content.

### Flux calculations by constraint-based modelling

A flux-balance model (Colombié *et al.*, 2015; Soubeyrand *et al.*, 2018) was constructed by integrating biochemical and physiological information about central metabolism dedicated to producing polyphenol compounds, and to breaking down and transforming extracellular nutrients to produce energy. Energy intermediates, both ATP and NAD(P)H, were explicitly considered and all the co-factors were defined as internal metabolites, which means that they were balanced, thus constraining the metabolic network not only through the carbon and nitrogen balance but also through the redox and energy status.

To solve the flux-balance model, constraints were applied for flux reversibility or irreversibility, and for out fluxes boundaries. Therefore, concentrations of accumulated metabolites and biomass components, expressed on a moles-per-fruit basis, were fitted and the derivative function calculated the corresponding fluxes. Stoichiometric network reconstruction encompassing central and polyphenol metabolism and their associated calculations were implemented using MATLAB (Mathworks R2012b, Natick, MA, USA) and the optimization toolbox solver quadprod with interior-point-convex algorithm for the minimization. Flux maps were drawn with the flux visualization tool of VANTED 2.1.0.

### Statistical analyses

For proteins, statistical analyses were conducted using the software Statistica 8 (TIBCO) based on the exported protein quantifications from Progenesis. We performed a principal component analysis (PCA, with NIPALS algorithm) to get an overview of the proteome data. We performed a partial least-squares analysis (PLS) (NIPALS algorithm) to differentiate proteins with a significant correlation to the time-points, the cultivar, or metabolite using all protein quantifications as continuous predictors (*x* matrix) and the time-points, cultivar, and quantified metabolite as dependent variables (*y* matrix). We applied a two-way ANOVA (*P*<0.05) to the selected proteins to determine their significance as affected by the time-point and cultivar, or the interaction between both.

All regressions were conducted in Microsoft Excel and based on the best fit *R*^2^. Pearson correlations between proteins or between proteins and selected metabolites or other variables were calculated using Statistica 8.

To integrate the different omics data, the protein inference and isoform redundancy issue was tackled by quantifying the proteins at the protein cluster level and EMPAI quantification (Scaffold_4.11.0, Proteome Software). To find the protein clusters that correlated to the modelled fluxes, we performed a two-block sparse partial-least-squares discriminant analysis (sPLS-DA) with the mixOmics package of R (Rohart *et al.*, 2017) using the DIABLO application (Singh *et al.*, 2019) with default parameters. We applied a threshold of *P*<0.001 (after false-discovery rate correction) for Pearson correlations in the relationships between the proteins and fluxes.

## Results and Discussion

### The proteome and metabolic profiles change significantly during fruit development

**Fig. 1.**
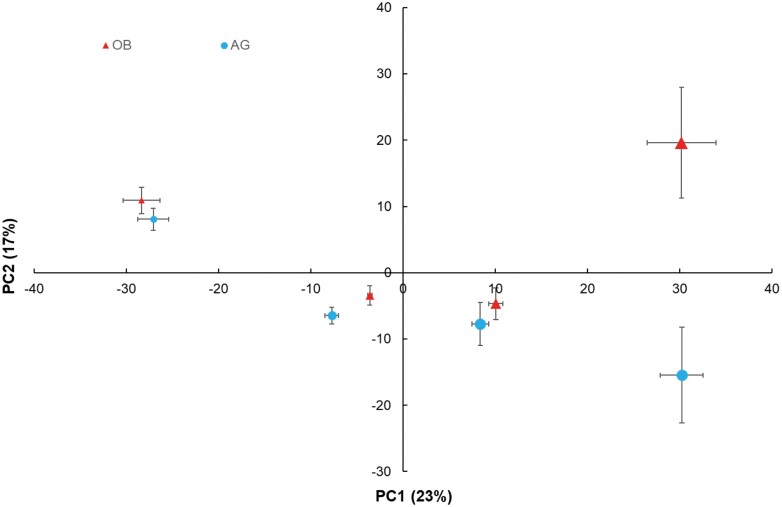
Principal component analysis of proteomics data for the fruit pulp of two cultivars of plantain banana during fruit development. Samples from the cultivars Agbaba (AG) and Obino l’Ewai (OB) were analysed at 6, 8, 10, and 12 weeks after bunch emergence, and the size of the data points is proportional to the time of sampling. The scores are means (±SE) per cultivar and time-point, *n*=4–5. A total of 2183 proteins were analysed (Supplementary Table S1).

As shown by unsupervised principal component analysis, the proteome differed significantly over time (Fig. 1; Supplementary Table S1). The first component explained 23% of total variability and was correlated with time, whilst the second component explained 17% of total variability and was correlated with the cultivar. A similar result was observed for the metabolite analysis (Supplementary Fig. S1).

For fruit growth, in both cultivars we observed a sigmoid curve (Supplementary Fig. S2). The growth pattern in banana is cultivar-dependent, and fertilization influences the growth and the shape of the fruit (Simmonds, 1953). A sigmoid type of growth has been described before in a triploid banana with a B genome (*M. balbisiana*, Awak legor) ­(Simmonds, 1953). The first period of fast growth is characterized by cell division and cell elongation, while the final period is due to elongation only (Ram *et al.*, 1962). The ­increase in cell number in the initiating region of the pulp has been reported to continue up to about 4 WAE in the cultivar Pisang lilin (a parthenocarp diploid *M. acuminata* with an AA genome) (Ram *et al.*, 1962). We found evidence to confirm the involvement of cell division in growth in our proteomics data. The development was undoubtedly dependent on the cultivar and the environment, but based on the abundance pattern of the identified histone proteins we deduce that cell division took place up to 8 WAE (Table 1); Supplementary Table S2). Histones are one of the primary components of chromatin and are synthesized during the S-phase. The speed of DNA replication depends on the rate of histone biosynthesis (Ma *et al.*, 2015).

**Table 1.** Proteins linked to growth in plantain banana pulp

Description	ANOVA *P*-value			No. peptides used for quantitation	Max fold-change	Highest mean abundance (WAE)	Lowest mean abundance (WAE)
	WAE	Cultivar	WAE×cultivar				
26S protease regulatory subunit 7	<0.01	0.87	0.94	6	1.7	6	12
26S proteasome non-ATPase regulatory subunit 1 homolog A	<0.01	0.01	0.01	7	2.9	6	12
26S proteasome regulatory subunit 6B homolog	<0.01	0.07	0.01	11	2.2	6	12
40S ribosomal protein S11	<0.01	0.92	0.45	8	3.0	6	12
40S ribosomal protein S13	<0.01	0.89	0.72	4	1.9	6	10
40S ribosomal protein S14	<0.01	0.40	0.79	9	3.2	6	12
40S ribosomal protein S16	<0.01	0.73	0.53	10	1.6	6	10
40S ribosomal protein S18	<0.01	0.13	0.03	14	1.8	6	12
40S ribosomal protein S19	<0.01	0.21	0.80	11	2.4	6	12
40S ribosomal protein S24-2	<0.01	0.54	0.54	3	3.7	6	12
40S ribosomal protein S25-4	<0.01	0.62	0.53	6	1.8	6	12
40S ribosomal protein S26-1	<0.01	0.08	0.70	3	2.0	6	12
40S ribosomal protein S3a	<0.01	0.48	0.34	8	2.0	6	12
40S ribosomal protein S5 (Fragment)	<0.01	0.06	0.32	7	2.5	6	12
40S ribosomal protein S9-2	<0.01	0.69	0.86	10	1.7	6	10
40S ribosomal protein SA	<0.01	0.75	0.17	8	3.5	6	12
50S ribosomal protein L12, chloroplastic	<0.01	0.28	0.86	2	4.7	6	12
60S acidic ribosomal protein P2B	<0.01	0.40	0.82	5	2.3	6	12
60S ribosomal protein L11	<0.01	0.43	0.13	6	2.6	6	12
60S ribosomal protein L12	<0.01	0.21	0.06	8	1.9	6	12
60S ribosomal protein L13-1	<0.01	0.55	0.82	8	1.9	6	10
60S ribosomal protein L22-2	0.01	0.88	0.14	2	2.0	6	12
60S ribosomal protein L23	<0.01	0.88	0.68	5	1.6	6	12
60S ribosomal protein L23A	<0.01	0.03	0.79	8	3.0	6	12
60S ribosomal protein L30	<0.01	0.97	0.58	2	2.3	6	12
60S ribosomal protein L34	0.01	0.69	0.45	5	2.5	6	12
60S ribosomal protein L35	<0.01	0.65	0.87	6	2.6	6	12
60S ribosomal protein L35a-3	<0.01	0.70	0.36	2	2.2	6	12
60S ribosomal protein L36-3	<0.01	0.06	0.65	6	2.9	6	12
60S ribosomal protein L37a	<0.01	0.46	0.79	2	10.8	6	12
60S ribosomal protein L4-1	<0.01	0.49	0.40	13	2.9	6	12
60S ribosomal protein L6	<0.01	0.42	0.60	7	2.1	6	10
60S ribosomal protein L7-2	<0.01	0.75	0.11	7	2.1	6	12
60S ribosomal protein L9	<0.01	0.57	0.00	6	2.4	6	12
Actin-depolymerizing factor 2	<0.01	0.05	0.12	2	3.9	6	12
Beta-glucosidase 1	<0.01	0.46	0.91	3	1.9	6	12
Eukaryotic initiation factor 4A-3	<0.01	0.19	0.63	24	1.7	6	12
Eukaryotic translation initiation factor 3 subunit I	<0.01	0.61	0.62	3	4.9	6	12
Guanine nucleotide-binding protein subunit beta-like protein	<0.01	0.13	0.14	11	2.6	6	12
Histone H2A.1	<0.01	0.07	0.09	2	9.7	6	12
Histone H2B	<0.01	<0.01	0.28	2	2.4	6	12
Histone H2B.6	<0.01	<0.01	0.92	6	4.5	6	12
Histone H4	<0.01	0.04	0.58	4	57.1	6	12
Proteasome subunit alpha type-1-A	<0.01	0.84	0.54	8	1.7	6	12
Proteasome subunit alpha type-2-A	<0.01	0.40	0.45	7	1.6	6	12
Proteasome subunit alpha type-5	<0.01	0.13	0.51	16	3.0	6	12
Proteasome subunit alpha type-6	<0.01	0.18	0.05	12	2.3	6	12
Proteasome subunit alpha type-7	<0.01	0.05	0.05	7	1.8	6	12
Proteasome subunit beta type-4	<0.01	0.26	0.77	5	4.1	6	10
Protein ASPARTIC PROTEASE IN GUARD CELL 1	<0.01	0.25	0.83	8	3.6	6	10
T-complex protein 1 subunit epsilon	<0.01	0.27	0.71	6	2.2	6	12
T-complex protein 1 subunit theta	<0.01	0.55	0.04	8	3.0	6	12
UDP-glucose 6-dehydrogenase 4	<0.01	0.13	0.02	5	3.0	6	12
UDP-glucuronic acid decarboxylase 6	<0.01	0.90	0.66	6	2.0	6	10

WAE: weeks after emergence.

This table only displays the ANOVA *P*-values of the protein paralog with the lowest value. The full list of significant protein paralogs can be seen in Supplementary Table S2.

Fast cell division and growth is also accompanied by high activity of cell-wall building and modifying enzymes (UDP-glucose 6-dehydrogenase, UDP-glucuronic acid decarboxylase, Beta-glucosidase), mRNA translation (eukaryotic initiation factors, ribosomal proteins), and protein folding (T-complex proteins) and turnover (proteasome complexes) (Table 1). The identified proteins involved in all these processes had their highest abundance at 6 WAE and significantly decrease in abundance at later stages (Table 1).

**Fig. 2.**
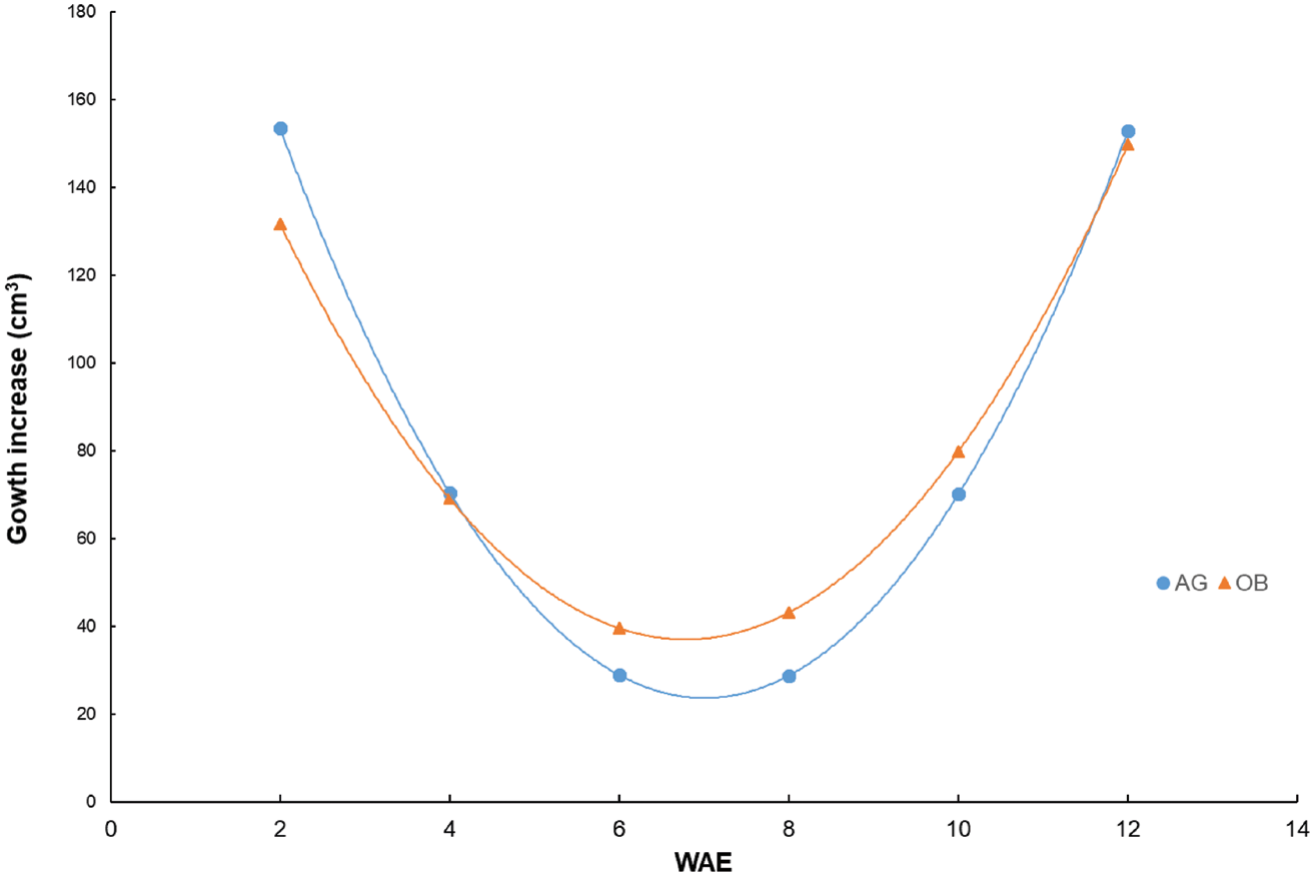
Calculated fruit growth rate of two plantain banana cultivars from 2–12 weeks after bunch emergence (WAE). AG, Agbaba; OB, Obino l’Ewai. The calculation of growth was derived from a cubic regression model of volume as a function of time (Supplementary Fig. S2).

**Fig. 3.**
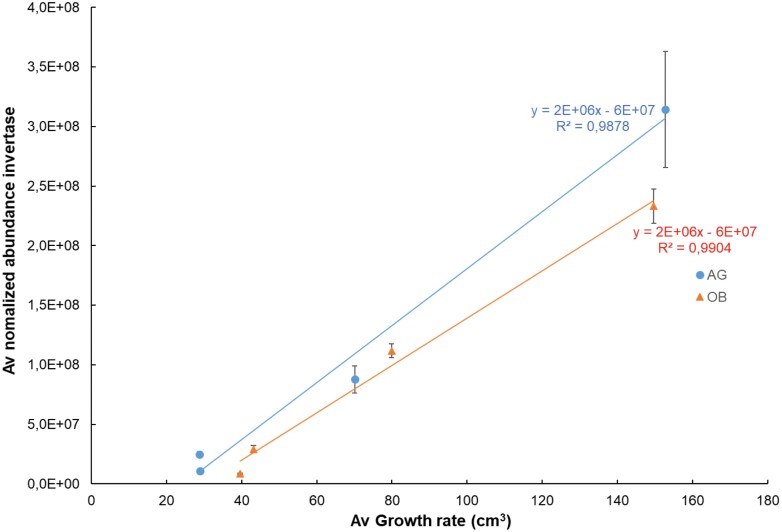
Correlations between calculated fruit growth rate (Fig. 2) and mean normalized abundance of cytoplasmic invertase (Mba10_g13890.1) for two plantain banana cultivars during the period 6–12 weeks after bunch emergence. . AG, Agbaba; OB, Obino l’Ewai. Data are means (±SE), *n*=4–5.

Banana pulp is a starch-synthesizing sink tissue that needs to get all its energy from sucrose unloaded from the phloem. From studies in tomato, it is known that the fruit growth ­consists of two phases: first, a period of rapid growth where sucrose synthase determines the sink strength, and second, a phase after the rapid growth has ceased, where invertase takes over (Nguyen-Quoc and Foyer, 2001). In the plantain bananas that we studied, we actually saw three growth phases: a fast growth phase that consisted of cell division and elongation (0–6 WAE), a phase of slow growth (6–8 WAE), and a final phase of fast growth (8–12 WAE) (Fig. 2). Based on their observed abundance patterns, we hypothesize that the initial fast growth phase was completely dominated by sucrose synthase (SuSy), while the final fast growth phase was dominated by invertase. The abundance of invertase showed a very strong correlation with the growth rate (Fig. 3).

### Starch metabolism: synthesis and breakdown take place simultaneously

On average across the two cultivars, the pulp at 6 WAE contained three times more fructose than glucose, but the concentration of fructose represented <1 % of that of starch and <15% of that of sucrose (Table 2). Among the hexose phosphates, the amount of G6P was 20-fold higher than G1P.

**Table 2.** Mean concentrations (µmol g^–1^ DW) of metabolites in plantain banana pulp during fruit development

Metabolite	Cultivar Agbagba (AG)						Cultivar Obino l’Ewai (OB)					
	Weeks after bunch emergence (WAE)						Weeks after bunch emergence (WAE)					
	2	4	6	8	10	12	2	4	6	8	10	12
Fructose 6-phosphate	0.5^D^	0.4^ABCD^	0.3^AB^	0.3^AB^	0.3^ABC^	0.5^CD^	0.5^CD^	0.4^ABCD^	0.3^A^	0.3^AB^	0.4^BCD^	0.4^ABCD^
Fructose	82.7^C^	51.5^BC^	19.1^AB^	9.5^A^	12.1^A^	9.7^A^	40.1^AB^	54.9^BC^	9.2^A^	8.3^A^	7.6^A^	4.1^A^
Glucose 1-phosphate	0.4^C^	0.3^ABC^	0.1^A^	0.2^AB^	0.2^ABC^	0.2^AB^	0.3^ABC^	0.3^BC^	0.1^A^	0.2^AB^	0.2^A^	0.2^AB^
Glucose 6-phosphate	3.6^BCDE^	2.6^ABC^	2.2^A^	2.5^AB^	3.4^BCDE^	4.5^E^	4.1^DE^	3.1^ABCD^	2.9^ABCD^	3.3^ABCDE^	3.8^CDE^	4.3^DE^
Glucose	39.8^B^	23.2^AB^	6.3^A^	4.2^A^	4.4^AB^	4.2^A^	21.2^AB^	21.8^AB^	3.7^A^	4.2^A^	4.1^A^	4.3^A^
Starch*	266.2^A^	313.6^ABC^	386.6^ABCD^	410.2^BCD^	457.4^D^	442.7^CD^	268.3^A^	264.2^A^	305.8^AB^	423.3^BCD^	449.1^D^	434^BCD^
Sucrose	92.1^ABC^	67.0^ABC^	75.5^AB^	108.3^BCD^	127.8^CDE^	160.2^E^	98.8^ABC^	68.6^A^	108.3^BCD^	141.6^DE^	156.1^E^	148.7^DE^

* Expressed as glucose equivalent.

Data for 2–10 WAE are means of five replicates; data for OB 12 WAE are means of three replicates. Different letters indicate significant differences among means as determined using two-way ANOVA followed by a Fisher LSD *post hoc* test (*P<0.05).

**Fig. 4.**
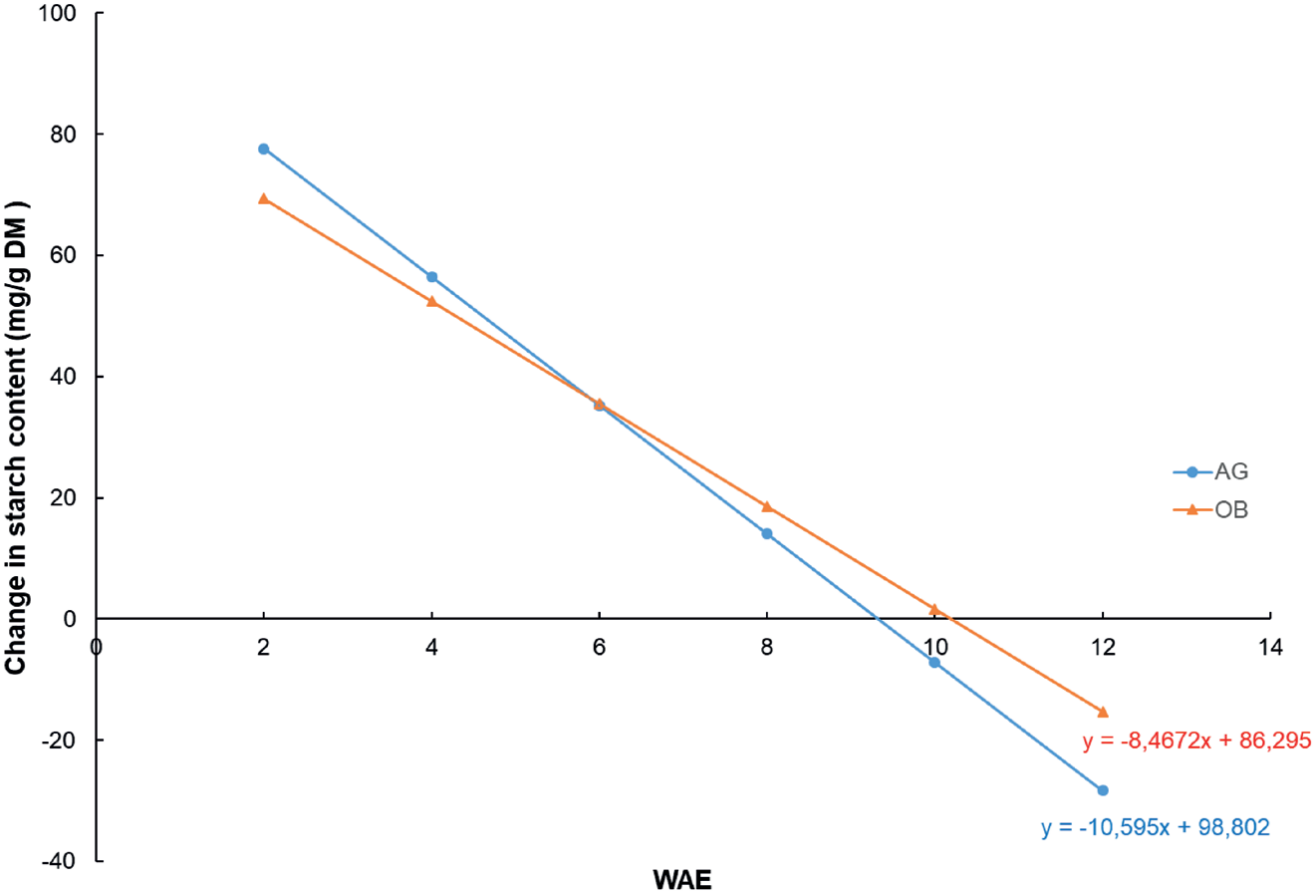
Calculated change in fruit starch content during development for two plantain banana cultivars. AG, Agbaba; OB, Obino l’Ewai. Samples harvested at 2–12 weeks after bunch emergence (WAE). The starch content was derived from a quadratic regression model as a function of time (Supplementary Fig. S3). Data are means of 3–5 replicates. Net starch breakdown is estimated to take place at 9.3 WAE and 10.2 WAE for AG and OB, respectively, and marks the end of maturation.

The accumulation of starch in the pulp cells was initially very rapid but showed a clear linear decline with time (Fig. 4). Starch synthesis and breakdown are processes that take place simultaneously, and the balance between them was clearly in favour of synthesis during the first 8–10 WAE, resulting in a net increase in starch content. During the net accumulation period, the plantain fruit accumulated on average up to 48% of their dry weight in starch (Supplementary Table S3).

#### Starch synthesis: identification of important precursors.

**Fig. 5.**
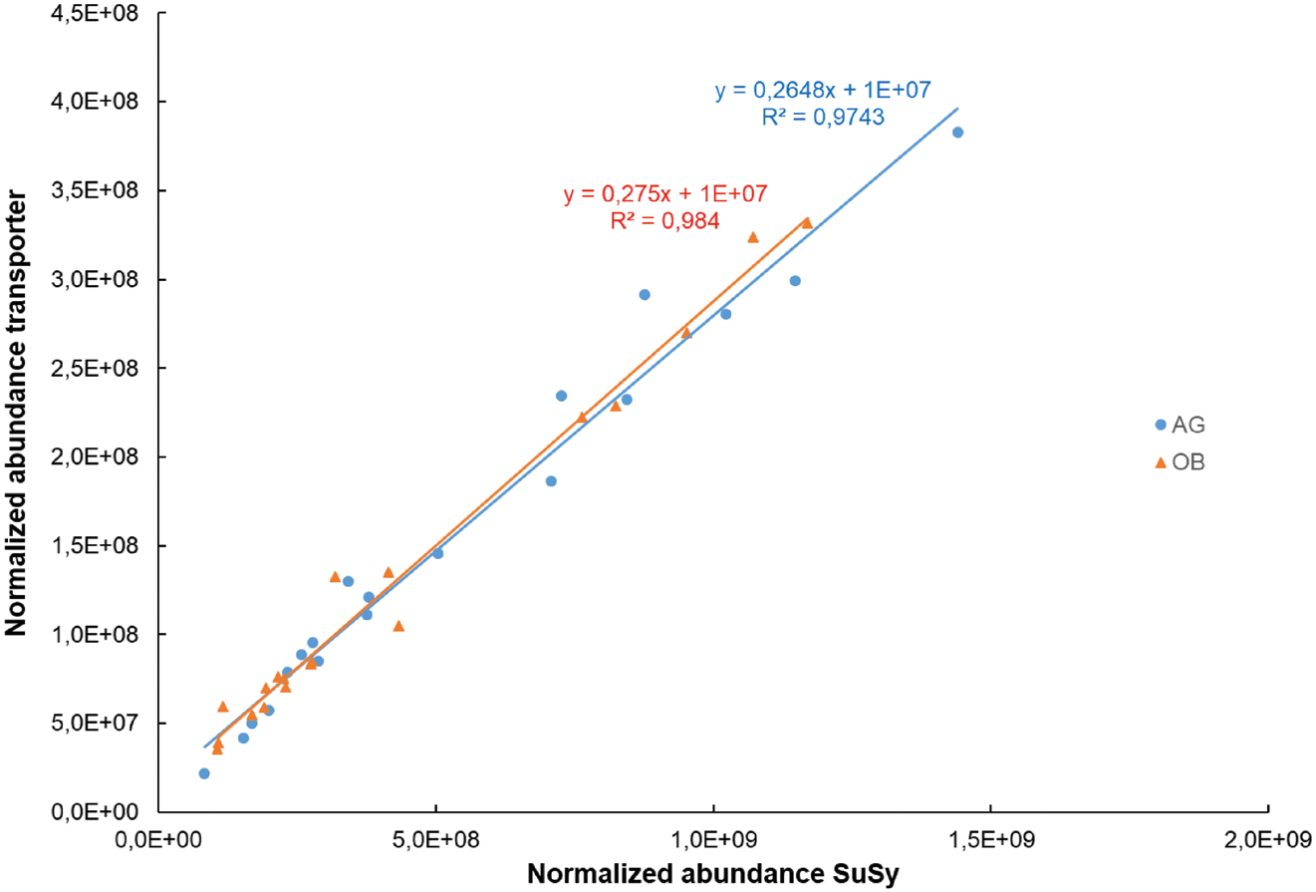
Correlations between the normalized abundances of the plastidic membrane transporter D-xylose-proton symporter-like 3 (Ma10_p26490) and sucrose synthase (SuSy, Ma08_p23180) in the fruit pulp of two plantain banana cultivars during the period 6–12 weeks after bunch emergence. AG, Agbaba; OB, Obino l’Ewai.

**Fig. 6.**
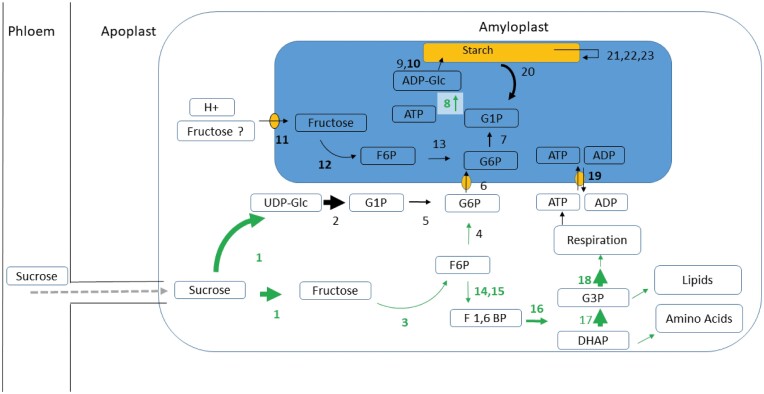
Net starch synthesis at 6 weeks after bunch emergence (WAE) based on proteomic data in the fruit pulp of two plantain banana cultivars. Enzyme numbers in bold were significantly higher in abundance at 6 WAE than later in development (Table 3). The arrows indicate the net direction of the fluxes. Enzymes and arrows in green were confirmed by the calculated fluxes (average of the two cultivars). The size of the arrow indicates the protein abundance, as determined by EMPAI quantification using Scaffold_4.11.0 (Proteome Software). Grey arrows indicate unidentified or unsure proteins. 1, sucrose synthase; 2, UTP-glucose-1-phosphate uridylyltransferase; 3, fructokinase; 4, glucose 6-phosphate isomerase, cytosolic; 5, phosphoglucomutase; 6, glucose 6-phosphate/phosphate translocator, chloroplastic; 7, phosphoglucomutase, chloroplastic; 8, glucose 1-phosphate adenylyltransferase large subunit 2, chloroplastic; 9, granule-bound starch synthase, chloroplastic/amyloplastic; 10, soluble starch synthase, chloroplastic/amyloplastic; 11, D-xylose-proton symporter-like 3, chloroplastic; 12, fructokinase; 13, glucose 6-phosphate isomerase; 14, ATP-dependent 6-phosphofructokinase; 15, pyrophosphate--fructose 6-phosphate 1-phosphotransferase subunit beta; 16, fructose-bisphosphate aldolase; 17, triosephosphate isomerase, cytosolic; 18, glyceraldehyde-3-phosphate dehydrogenase, cytosolic; 19, ADP,ATP carrier protein, chloroplastic; 20, alpha-1,4 glucan phosphorylase L isozyme, chloroplastic/amyloplastic; 21, phosphoglucan, water dikinase, chloroplastic; 22, phosphoglucan phosphatase LSF1, chloroplastic; 23, isoamylase 3, chloroplastic. All enzymes are listed in Supplementary Table S2, while Table 3 lists only those that showed significant correlations with either starch or sucrose.

Because starch synthesis in fruit pulp is confined to amyloplasts, it relies entirely on translocation of metabolites from the cytosol through the amyloplast envelope. The form in which carbon enters the amyloplast has long been a matter of debate (Hofius and Börnke, 2007). The triose phosphate transporter from chloroplasts is a well-annotated and highly studied transporter in the plastid envelope of many plants; however, there is discussion as to whether the genes are expressed in non-green tissue (Tobias *et al.*, 1992; Neuhaus and Emes, 2000). In potato it is clear that triose phosphate is not the substrate taken up to support starch synthesis (Hofius and Börnke, 2007). Our data also pointed to the same conclusion since we were not able to identify a triose phosphate transporter protein in plantain pulp. Amyloplasts of tubers or fruits are also normally not able to generate hexose phosphates from C3 compounds due to the absence of fructose 1,6-bisphosphatase activity (Nguyen-Quoc and Foyer, 2001; Hofius and Börnke, 2007), and they rely on the import of cytosolically generated hexose ­phosphates as the source of carbon for starch biosynthesis (Entwistle and Rees, 1988; Hofius and Börnke, 2007). This also seems to be the case in plantain since we were not able to identify a fructose 1,6-bisphosphatase protein during the time-course of our study. The enzyme does seem active though in non-photosynthetic tissues where it controls the rate of F6P production in the gluconeogenic pathway (Hofius and Börnke, 2007). We have previously identified the enzyme when we analysed ripening in detached fruits, but only in low quantities (Bhuiyan *et al.*, 2020), so it might play a role in banana in starch breakdown much later when the ripening and sugar synthesis is more advanced. None of the three predicted adenine nucleotide BT1 transporters (Ma10_p26970, Ma07_p09880, Ma06_p06780) that transport ADP-glucose across the plastid membrane was identified in our present study. Therefore, it is also unlikely that ADP-glucose moves across the amyloplast envelope to provide substrates for starch synthesis. We suggest that in plantain banana the cytosolic glucose 6-phosphate is an important direct source for starch synthesis, as is the case in maize (Tobias *et al.*, 1992). This was also confirmed by the high abundance of the glucose 6-phosphate/phosphate translocator (6), phosphoglucomutase (5), and the glucose 1-phosphate uridylyltransferase (2) (as numbered in Table 3). We identified a so far uncharacterized sugar translocator (11, D-xylose-proton symporter-like 3; Ma10_p26490.1) that had an almost perfect correlation (*P*<0.0001, *R*=0.99) with SuSy (1) (Fig. 5). Plastids are able to transport sugars across their membranes (Patzke *et al.*, 2019); however, only two plastidic sugar transporters are well known and described (Weber *et al.*, 2000; Niittylä *et al.*, 2004). These transporters reside in the inner envelope membrane and mediate the export of maltose and glucose (Cordenunsi-Lysenko *et al.*, 2019). Given the tight correlation that we observed with SuSy, we hypothesize that Ma10_p26490.1 transports fructose across the amyloplast membrane. The abundance pattern of the plastidic fructokinase (12) supports this hypothesis (Table 3; Fig. 6). Only very few reports are available on plastid fructose/glucose/sucrose H transporters (Patzke *et al.*, 2019), and hence more studies are needed to confirm their physiological roles in starch synthesis.

**Table 3.** Proteins linked to starch and sugar metabolism in plantain banana pulp and their correlations with starch and sucrose

Enzyme ID no.	Description	ANOVA *P*-value			No. peptides used for quantitation	Max fold-change	Highest mean abundance (WAE)	Lowest mean abundance	*R*	
		WAE	Cultivar	DAF×cultivar					Starch	Sucrose
1	Sucrose synthase	<0.01	0.03	0.59	10	8.2	6	12	**–0.41**	**–0.68**
2	UTP-glucose-1-phosphate uridylyltransferase	<0.01	0.42	0.07	30	2.1	12	6	0.16	**0.50**
3	Fructokinase-1	<0.01	0.26	0.83	9	2.0	6	12	–0.28	**–0.56**
4	Glucose 6-phosphate isomerase, cytosolic 1	<0.01	0.05	0.19	12	9.0	12	6	0.21	**0.44**
5	phosphoglucomutase, putative, expressed	0.06	0.47	0.55	7	8.2	12	8	0.12	0.21
6	Glucose 6-phosphate/phosphate translocator 2, chloroplastic	<0.01	0.55	0.45	5	3.0	12	6	0.02	0.29
7	Phosphoglucomutase 2C chloroplastic	<0.01	0.40	0.94	24	1.9	12	6	0.21	**0.60**
8	Glucose 1-phosphate adenylyltransferase large subunit 2, chloroplastic	<0.01	0.55	0.35	14	2.8	6	12	**–0.43**	**–0.67**
9	Granule-bound starch synthase 1, chloroplastic/amyloplastic	0.02	0.65	0.45	4	2.4	12	6	0.27	0.23
10	Soluble starch synthase 3, chloroplastic/amyloplastic	0.03	0.29	0.30	11	3.2	6	12	0.13	**–0.34**
11	D-xylose-proton symporter-like 3, chloroplastic	<0.01	0.06	0.58	4	6.2	6	12	**–0.40**	**–0.67**
12	Probable fructokinase-6, chloroplastic	0.04	0.12	0.29	3	1.7	6	12	–0.03	–0.22
13	Glucose 6-phosphate isomerase 1, chloroplastic	0.06	0.32	0.25	13	1.3	12	10	0.23	**0.50**
14	ATP-dependent 6-phosphofructokinase 3	0.01	0.45	0.51	5	1.5	6	12	–0.12	**–0.53**
15	Pyrophosphate-fructose 6-phosphate 1-phosphotransferase subunit alpha	<0.01	0.42	0.78	4	2.0	6	10	**–0.39**	**–0.52**
16	Fructose-bisphosphate aldolase cytoplasmic isozyme	<0.01	0.01	0.68	28	1.3	6	12	–0.27	**–0.39**
17	Triosephosphate isomerase, cytosolic	<0.01	0.01	0.08	3	2.7	12	6	0.05	0.31
18	Glyceraldehyde-3-phosphate dehydrogenase 2, cytosolic	<0.01	0.18	0.13	25	1.6	6	10	**–0.39**	–0.17
19	ADP,ATP carrier protein 1, chloroplastic	<0.01	0.23	0.03	3	72.7	6	12	**–0.44**	**–0.57**
20	Alpha-1,4 glucan phosphorylase L isozyme, chloroplastic/amyloplastic	<0.01	0.89	0.29	92	3.3	12	6	0.22	**0.64**
21	Phosphoglucan, water dikinase, chloroplastic	<0.01	0.57	0.99	28	3.5	12	6	0.15	**0.52**
22	Phosphoglucan phosphatase LSF1, chloroplastic	<0.01	0.26	0.96	6	1.8	12	6	0.03	**0.43**
23	Isoamylase 3, chloroplastic	<0.01	0.15	0.92	14	2.5	12	6	0.13	**0.51**
24	4-alpha-glucanotransferase	<0.01	0.29	0.00	32	2.3	12	6	0.18	**0.68**
25	Plastidic glucose transporter	<0.01	0.66	0.10	4	2.4	10	6	0.19	0.13
26	Soluble inorganic pyrophosphatase, chloroplastic	<0.01	0.29	0.17	7	3.3	12	6	0.12	**0.34**
27	Sucrose-phosphate synthase	0.01	0.93	0.90	25	2.0	12	6	0.15	**0.45**
28	Pyrophosphate-energized vacuolar membrane proton pump	<0.01	0.05	0.23	20	5.6	12	6	0.21	**0.46**
29	Beta-fructofuranosidase 2C insoluble isoenzyme 3	<0.01	0.54	0.42	17	37.3	12	6	0.11	**0.41**
30	Monosaccharide-sensing protein	<0.01	0.69	0.91	3	9.7	12	6	0.20	**0.50**
31	Beta-fructofuranosidase, insoluble isoenzyme 3	<0.01	0.82	0.82	5	19.5	12	6	0.11	**0.41**

WAE, weeks after bunch emergence. This table only displays the ANOVA *P*-values of the protein paralog with the lowest value. The full list of significant protein paralogs can be seen in Supplementary Table S2. *R* values are Pearson correlation coefficients. Correlations in bold are significant at *P*<0.05.

Following the uptake of glucose 6-phosphate into the pulp amyloplast, starch synthesis starts via the concerted actions of phosphoglucomutase (7), glucose 1-phosphate adenylyltransferase (8), and the starch polymerizing reactions (9, 10) (Table 3; Fig. 6). In case of fructose, the action of fructokinase (12) and glucose 6-phosphate isomerase (13) are required. Soluble starch synthase (10) decreased in abundance during development while granule-bound starch synthase increased in abundance (9) (Table 3), suggesting that the former is more important during early synthesis. The fact that the polymerizing reactions of starch synthesis were not dominant in the control of accumulation was related to the balance between sink strength, starch synthesis, and starch breakdown, and has been observed before (Tetlow *et al.*, 2004). On the basis of ANOVA and correlation analyses, the main drivers of starch synthesis in the plantain pulp seemed to be sucrose synthase (1), glucose 1-phosphate adenylyltransferase (ADP-glucose pyrophosphorylase, AGPase) (8), ADP,ATP carrier protein (19), and the so far uncharacterized membrane sugar transporter (11) (Fig. 6; Table 3). Beyond their role as intermediates in the conversion of sucrose to starch, hexose phosphates also serve as substrates for glycolysis and the oxidative pentose phosphate pathway. The significant correlations with starch observed for pyrophosphate-fructose-6-phosphate 1-phosphotransferase (15) and glyceraldehyde-3-phosphate dehydrogenase (18) (Table 3) was probably due to their functions in glycolysis. Whereas in chloroplasts the ATP necessary for starch synthesis is provided through photosynthesis, in pulp the amyloplasts have to import ATP originating from respiration via the cytosol through an ADP,ATP transport protein (19). This enzyme was highly abundant when starch synthesis was high (Table 3; Fig. 6).

#### Starch breakdown: enzymes that initiate breakdown.

On average across the two cultivars, the pulp at 12 WAE contained twice as much fructose as glucose, but the concentration of fructose represented <0.5 % of that of starch and <5% of that of sucrose (Table 2). Among the hexose phosphates, the amount of G6P was 20-fold higher than G1P.

Starch-to-sucrose metabolism has been extensively studied in model systems in the context of energy sources for plant growth and development (Streb and Zeeman, 2012); however, the breakdown of starch in fleshy fruits such as bananas is less well understood (Cordenunsi-Lysenko *et al.*, 2019). All the genes involved in starch breakdown have been mapped on the banana genome (Xiao *et al.*, 2018). Based on what is known from Arabidopsis, it has been hypothesized in banana that starch-phosphorylating enzymes, termed glucan water dikinases (GWDs), phosphorylate the C6 position and that the phosphoglucan water dikinase (PWD) phosphorylates the C3 position of the glycosyl residues in starch (Cordenunsi-Lysenko *et al.*, 2019). The role of phosphorylases including GWDs and PWD in starch breakdown during banana ripening is less well understood, but phosphorylation at the C3 and C6 position of the glucosyl residues in the starch of freshly harvested unripe bananas has already been found, as well as the presence of PWD and GWDs (Cordenunsi-Lysenko *et al.*, 2019). The steric hindrance of these phosphorylated groups alters the organization of the granule and it has been hypothesized that PWD acts downstream of GWDs, and that the induced phosphorylation of banana starch favours granule hydration and phase transition from the crystalline state to the soluble state (Cordenunsi-Lysenko *et al.*, 2019). Our results confirmed that dikinases played a role in early starch breakdown but not that PWD acts downstream of GWDs. We identified the sole PWD protein present in the banana genome (21) (Ma09_p07100.1;Mba09_g06570.1) as being present at the early stage of the starch breakdown process and as being significantly up-regulated (Table 3), while neither of the two GWD proteins could be detected. We have previously identified GWD1 during the ripening of detached plantain fruits (Bhuiyan *et al.*, 2020), and Xiao *et al.* (2018) identified it as being expressed at the late ripening stages in detached fruits.

**Fig. 7.**
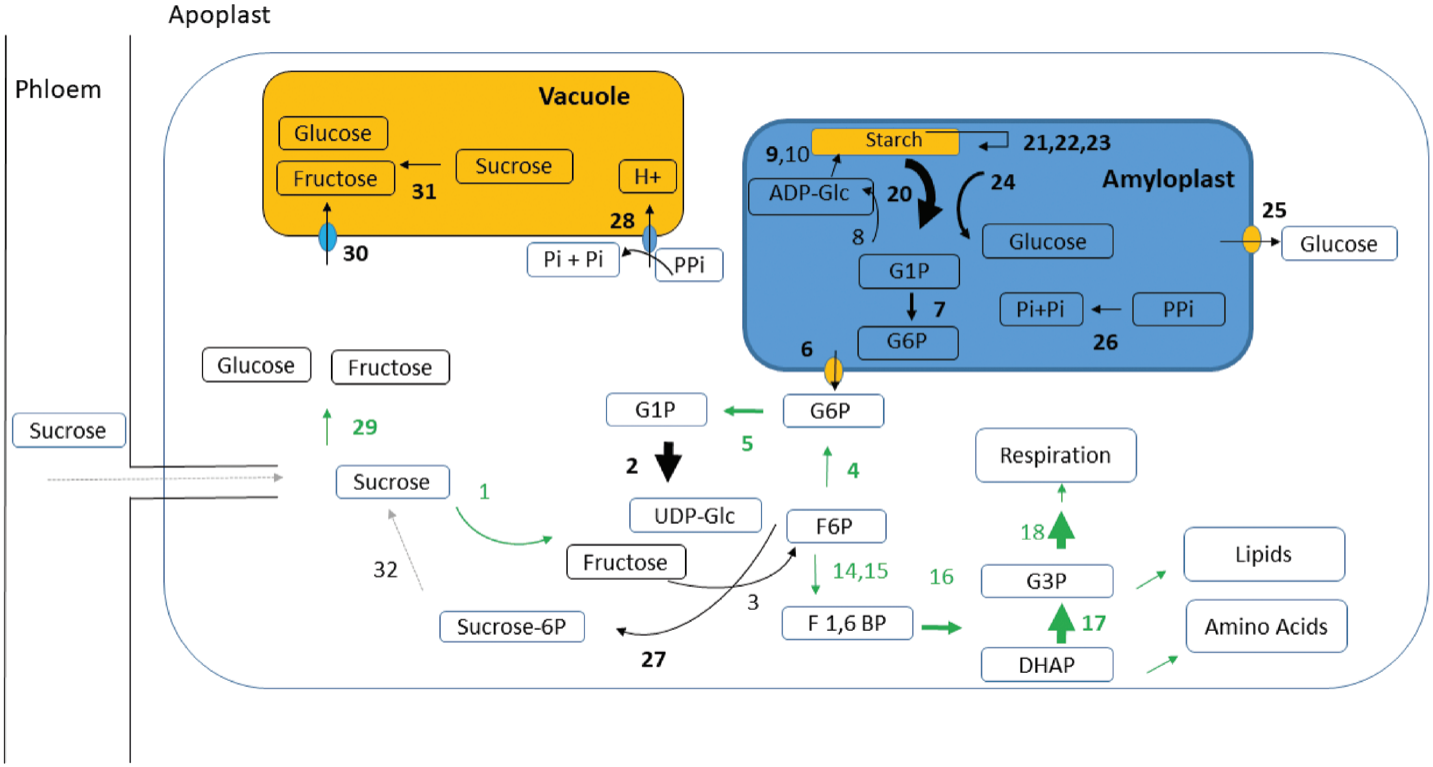
Net starch breakdown at 12 weeks after bunch emergence (WAE) based on proteomic data in the fruit pulp of two plantain banana cultivars. Enzyme numbers in bold were significantly higher in abundance at 12 WAE than earlier in development (Table 3). The arrows indicate the net direction of the flux is indicated. Enzymes and arrows in green were confirmed by the calculated fluxes (average of the two cultivars). The size of the arrow indicates the protein abundance, as determined by EMPAI quantification using Scaffold_4.11.0 (Proteome Software). Grey arrows indicate unknown or unsure proteins. Enzyme numbers are the same as listed in Fig. 6. Additional enzymes are: 24, 4-alpha-glucanotransferase disproportioning enzyme; 25, plastidic glucose transporter; 26, soluble inorganic pyrophosphatase, chloroplastic; 27, sucrose-phosphate synthase; 28, pyrophosphate-energized vacuolar membrane proton pump; 29, invertase; 30, monosaccharide-sensing protein; 31, invertase; 32, sucrose-phosphatase (identification unsure, only one peptide). All enzymes are listed in Supplementary Table S2, while Table 3 lists only those that showed significant correlations with either starch or sucrose.

Phosphorolytic cleavage seems to be one of the first starch breakdown reactions and this was corroborated by the abundance profiles of phosphorylase (20) and the G6P transporter (6) (Table 3; Fig. 7). An increase in abundance and activity of phosphorylase has also been observed previously during maturation and ripening (Da Mota *et al.*, 2002). Other enzymes also appeared to contribute to the early degradation of starch, with phosphoglucan phosphatases (22), the α-1,6-glucosidase starch debranching enzyme (DBE) (23), and 4-α-glucanotransferase disproportionating enzymes (DPEs) (24) increasing significantly in abundance with time (Table 3; Fig. 7). We also observed increased abundance of the plastidic glucose transporter (25), while the maltose excess protein transporter was not detected. Since we also did not detect either alpha or beta-amylases at this early stage of ripening, we hypothesize that they act later in the process. Previous studies of detached ripening fruits found that plastidic alpha amylase acts before beta amylase (Purgatto *et al.*, 2001; Bhuiyan *et al.*, 2020). Beta amylase is essential to complete the breakdown, and its up-regulation was reported to be correlated with a decrease in starch during ripening.

### Sucrose synthesis: competition between vacuolar storage and recycling for growth and starch re-synthesis

The starch breakdown products G1P and glucose are converted to sucrose, which can then be metabolized further. The enzymes with the highest correlations with sucrose were DPE (24), alpha-1,4 glucan phosphorylase (20), and chloroplastic phosphoglucomutase (7) (Table 3). UTP-glucose-1-phosphate uridylyltransferase (2) was one of the most abundant proteins in the pulp and its abundance increased with time (Table 3; Fig. 6). The production of UDP-glucose could lead to sucrose synthesis either through SuSy (1), which was still abundantly present, or through sucrose-phosphate synthase (27), which had its highest abundance at 12 WAE (Table 3; Fig. 7). We did not confidently identify sucrose phosphatase at this early stage of ripening. Only one peptide was found with low confidence, which was probably due to the low abundance of the enzyme. We did identify sucrose phosphatase in our previous study of detached ripening fruits; it had low abundance and was significantly up-regulated in the very late ripening stages (Bhuiyan *et al.*, 2020). The sucrose that was formed could have been degraded by invertase (29) and/or SuSy (1) in the cytoplasm, or could have been transported to the vacuole for storage or further processing by invertase (31) (Table 3, Fig. 7). Invertase (29; Mba10_g13890.1) is only encoded on the B genome and is predicted to be localized in the cytoplasm according to the DeepLoc 1.0 software (Almagro Armenteros *et al.*, 2017). The cytoplasmatic homologue coded on the *M. acuminata* genome (A genotype) is most probably either not expressed or expressed at only a very low level since we did not find a confident specific spectrum. We have previously shown that invertase is more abundant in plantain compared with a Cavendish type banana (Bhuiyan *et al.*, 2020), and higher invertase activity in cooking bananas has been associated with an altered sucrose/(glucose + fructose) ratio (Fils-Lycaon *et al.*, 2011). The breakdown of sucrose in the cytoplasm by invertase would enable an energy flow back to starch synthesis and glycolysis to support further growth, as discussed above (Fig. 3). Plantains are indeed a lot bigger than dessert bananas and contain much more starch. Part of the metabolized sucrose is most likely also transported to the vacuole since we identified a monosaccharide transporter (30) (Ma04_p22640.1;Mba04_g23280.1) that had its highest abundance at 12 WAE (Table 3; Fig. 7). DeepLoc predicted its location as the plasma membrane with a likelihood of 0.49 and as the vacuole with a likelihood of 0.35. Since no cell wall invertase was identified and since we did identify invertase in the cytoplasm, we assume that the monosaccharide transporter is located in the vacuolar membrane (Fig. 7). The up-regulation of the vacuolar pyrophosphate energized proton pump (28) (Ma07_p22370.1) (Table 3) would also facilitate the transport of sugars across the vacuolar membrane (Maeshima, 2000). Alterations in inorganic pyrophosphate (PPi) metabolism have a strong effect on sugar metabolism, with higher PPi levels increasing starch accumulation and decreasing the level of sucrose. Decreased PPi levels have been associated with lower starch biosynthetic rates (Osorio *et al.*, 2013). The overexpression of a pyrophosphatase in tomato results in an increase in the major sugars, a decrease in starch, and an increase in vitamin C (ascorbic acid) (Osorio *et al.*, 2013). We observed an increased abundance of soluble inorganic pyrophosphatase in the amyloplast (26) and in the vacuole (28) that coincided with the decrease in starch synthesis and increase in sucrose (Tables 2, 3; Fig. 7), and proteins linked to ascorbate synthesis (GDP-mannose 3,5-epimerase) (Wolucka and Van Montagu, 2003) and anti-oxidant defense (ascorbate peroxidase, monodehydroascorbate reductase) also had their highest abundances at 12 WAE (Table 4). Ascorbic acid is also a co-factor of 1-aminocyclopropane-1-carboxylic acid oxidase (ACO) that catalyses the final step in the biosynthesis of the plant hormone ethylene (Smith *et al.*, 1992).

**Table 4.** Proteins linked to ascorbate synthesis, anti-oxidant defence, and ethylene responses in plantain banana pulp, and their correlations with ACO

Description	ANOVA *P*-value			No. peptides used for quantitation	Max fold-change	Highest mean abundance (WAE)	Lowest mean abundance (WAE)	*R*	
	WAE	Cultivar	WAE×cultivar					Sucrose	ACO
1-aminocyclopropane-1-carboxylate oxidase	<0.01	0.30	0.03	39	19.1	12	6	**0.60**	**1.00**
Sorbitol dehydrogenase	<0.01	0.42	0.02	13	3.4	12	6	**0.63**	**0.91**
Germin-like protein 12-1	<0.01	0.06	<0.01	5	123.8	12	6	**0.58**	**0.88**
Pectinesterase/pectinesterase inhibitor PPE8B	<0.01	0.34	0.01	18	12.8	12	6	**0.59**	**0.84**
Putative Pectinesterase	<0.01	0.86	0.39	11	7.1	12	6	**0.57**	**0.82**
L-ascorbate peroxidase, cytosolic	<0.01	0.07	<0.01	14	2.5	12	6	**0.57**	**0.82**
GDP-mannose 3,5-epimerase 1	<0.01	0.21	0.56	13	1.9	12	6	**0.50**	**0.82**
Probable L-ascorbate peroxidase 7, chloroplastic	<0.01	0.12	0.87	7	3.4	12	6	**0.50**	**0.75**
Lichenase	<0.01	0.23	<0.01	20	46.1	12	6	**0.56**	**0.73**

WAE, weeks after bunch emergence. *R* values are Pearson correlation coefficients. This table only displays the ANOVA *P*-values of the protein paralog with the lowest value. The full list of significant protein paralogs can be seen in Supplementary Table S2. Correlations in bold are significant at *P*<0.05.

### Cultivar-specific ethylene biosynthesis and auxin scavenging

The increase in sucrose production was significantly correlated with the abundance of 1-aminocyclopropane-1-carboxylate oxidase (ACO) (Table 4), the enzyme that produces ethylene. The critical point during ripening is when the tissue becomes more sensitive to ethylene and the internal concentration reaches the threshold required to induce biological responses (Paul *et al.*, 2012). Banana has two interconnected ethylene feedback loops (Lü *et al.*, 2018): the first is a positive one that is dependent on NAC transcription factors, while the second is controlled by MADS transcription factors and is able to maintain ethylene synthesis even when the first loop is blocked. It has been shown that banana ACO has a NAC motif in the promoter sequence (Lü *et al.*, 2018) and that ripening is a highly coordinated process regulated at the transcript level (Kuang *et al.*, 2021). We observed a significant interaction between cultivar and WAE for ACO abundance (Table 4). The disappearance of the large pool of starch in favour of accumulation of soluble sugars is known to contribute to pulp softening (Shiga *et al.*, 2011). We found that a putative pectinesterase-related protein and a lichenase were correlated with both sugar and ACO (Table 4), and these enzymes are associated with pulp softening (Li *et al.*, 2019; Bhuiyan *et al.*, 2020). Proteins with sequence similarity to germins have been identified in various plant species. These ‘germin-like proteins’ (GLPs) have a global low sequence identity with germins, and constitute a large and highly diverse family with various functions, among which is auxin binding (Bernier and Berna, 2001). Two auxin-correlated GLPs have been isolated in plum and are correlated with changes in levels of autocatalytic ethylene and associated ripening (El-Sharkawy *et al.*, 2010). Differential expression was found in two contrasting cultivars and it was hypothesized that the different endogenous auxin levels altered the levels of available ethylene, hence resulting in the different ripening phenotypes. We found a GLP (12-1) that had a significant correlation with ACO (Table 4). A Prosite scan indicated that the protein has a Fe(^2+^) 2-oxoglutarate dioxygenase domain profile. A 2-oxoglutarate-dependent-Fe(^2+^) dioxygenase in rice has been shown to convert active auxin (indole acetic acid) into biologically inactive 2-oxoindole-3-acetic acid, supporting a key role in auxin catabolism (Zhao *et al.*, 2013). Moreover, the protein also showed a significant cultivar × WAE interaction, which was associated with the earlier ripening of the Agbagba cultivar (Table 4). We hypothesize that this GLP/2-oxoglutarate dioxygenase would catabolize auxin and hence stimulate ripening. In banana, it has been shown that ethylene promotes ripening and auxins delays it (Purgatto *et al.*, 2001; Mainardi *et al.*, 2006; Kuang *et al.*, 2021), and the same has also been shown in papaya (Zhang *et al.*, 2020).

In plum, it has been shown that ethylene is a crucial factor that affects overall sugar metabolism, stimulating sorbitol breakdown via increased sorbitol dehydrogenase (Farcuh *et al.*, 2018). We also observed a strong correlation between ACO and a sorbitol dehydrogenase, which catabolizes sorbitol into fructose. There was a significant cultivar × WAE interaction, with the Agbagba having a significantly earlier response than Obino l’Ewai (Table 4).

### Metabolite fluxes generally decrease during fruit development

**Fig. 8.**
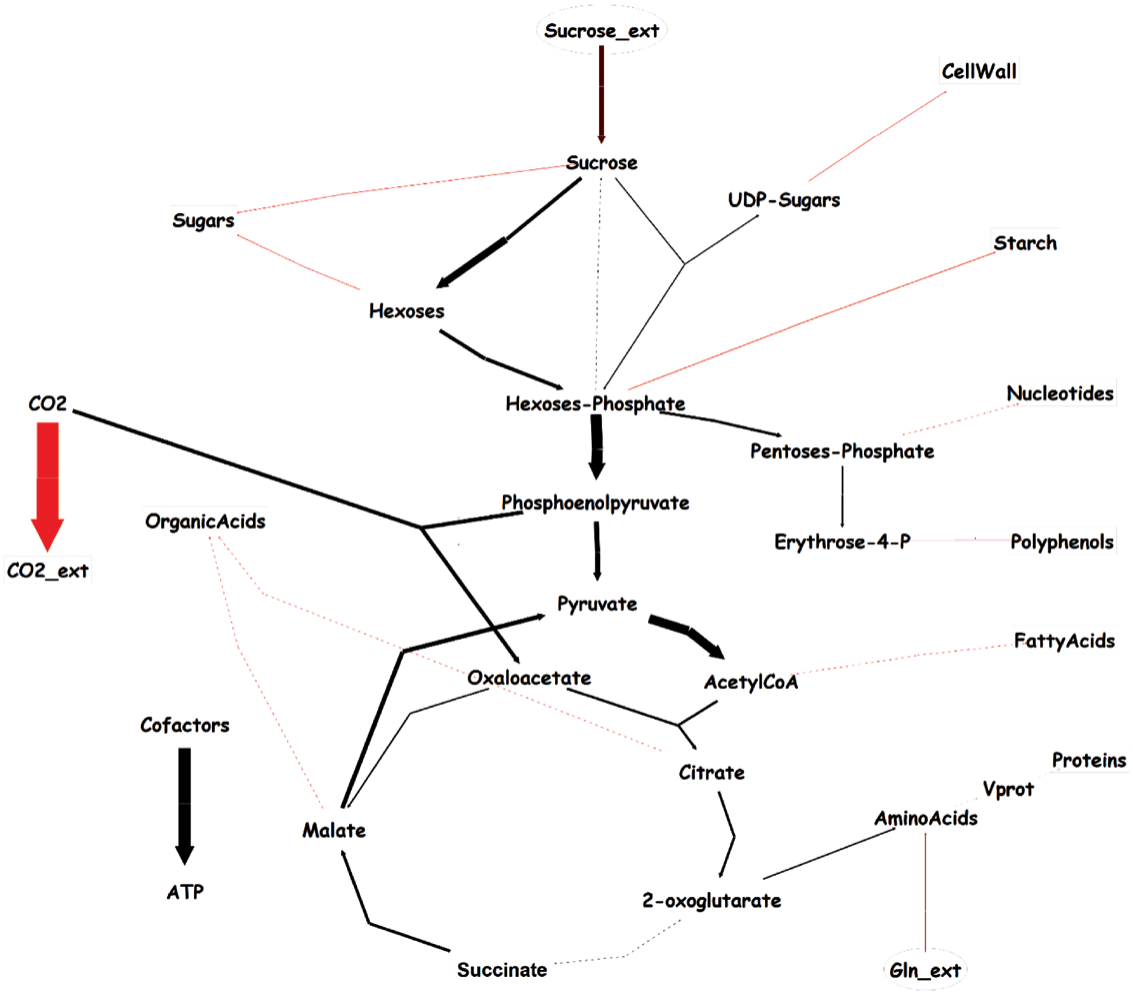
Simplified diagram of fluxes in the plantain banana cultivar Agbaba at 2 weeks after bunch emergence. The widths of the arrows are proportional to the activity of the flux. The fluxes were determined using constraint-based modelling from fluxes calculated on a gram DW basis (see Supplementary Table S4), and show high activities for fluxes in glycolysis, the TCA cycle, and in particular respiration (highlighted in red). A similar pattern was found for the Obino l’Ewai cultivar (Supplementary Fig. S4).

The concentrations of metabolites in the pulp determined from 2–12 WAE (Table 2) were fitted to calculate corresponding fluxes used as constraints in a metabolic model (Supplementary Table S4). We selected three stages for detailed examination, 2, 6, and 12 WAE, and the highest activities were observed for fluxes involved in respiration, glycolysis, and the TCA cycle (Supplementary Fig. S4; Supplementary Table S4). These fluxes had their highest activity at 2 WAE (Fig. 8) and a global decrease during fruit development was seen in both cultivars, in agreement with metabolic fluxes described in tomato fruit (Colombié *et al.*, 2015). We hypothesise that high respiration is associated with the cell division that occurs during the first growth phase, and this is followed by a global decrease in flux activity during the second and third-growth phases, when only elongation takes place. No increase in respiration was detected at the end of the maturation period, probably because at 12 WAE the fruit that we examined were still in the early maturation phase and the burst had not yet taken place.

The flux analyses at 6 WAE and 12 WAE complemented the proteome data, and good concordance was observed for the major reactions in starch biosynthesis (Fig. 6): sucrose synthase (1), fructokinase (3), glucose 6-phosphate isomerase (4), and glucose 1-phosphate adenylyltransferase (8). For the fluxes at 12 WAE in the starch degradation pathway (Fig. 7), in addition to invertase (29), the fluxes through sucrose synthase (1) and glucose 6-phosphate isomerase (4) also pointed towards a net cleavage of sucrose. Some uncertainties in the flux calculations might be attributable to the assumptions required to solve the model (flux minimization).

### Conclusions

By combining proteomics and flux studies, our study provides unique insights into the order of appearance and dominance of specific enzymes/fluxes involved in starch and sugar synthesis and breakdown in plantain. Our results for fluxes give a broader analysis of metabolism. Although the fluxes were calculated in a non-compartmented network, we have shown that in conjunction with proteome data they can give a satisfactory picture of the dynamics of metabolism during fruit development. Maturation in plantain is completed around 10 weeks after bunch emergence, as indicated by a net breakdown in starch. The import of G6P and possibly fructose into the amyloplast are the main drivers of starch synthesis. Soluble starch synthase probably plays a more important role in starch synthesis during the early fruit development period, while granule-bound starch synthase most likely influences starch at the mature stage. For starch breakdown, DPE and phosphorylase mainly produce the first hexoses for sugar synthesis and amylases come into play at a later stage in ripening. Cytoplasmic invertase in plantain seems to play an important role in the breakdown of sucrose to support further growth and to maintain a high starch content. Our data point towards an interplay between auxins and ethylene controlling the ripening process. Despite the fact that both the plantain cultivars that we studied are extremely close genetically, we still found significant differences in ripening. The earlier ripening in Agbagba might be related to an earlier induction of the second ethylene system and a greater scavenging of auxins. Overall, our results contribute to a better understanding of fruit development and maturation in banana, and more specifically in plantains.

## Supplementary data

The following supplementary data are available at *JXB* online.

Fig. S1. Principal component analysis of the metabolic compounds.

Fig. S2. Changes in fruit volume over time for the two plantain varieties.

Fig. S3. Changes in starch contents over time for the two plantain varieties.

Fig. S4: Simplified flux maps at 2, 6, and 12 WAE.

Table S1. All proteins identified in fruit pulp.

Table S2. Proteins with differential abundance during development.

Table S3. Concentrations of soluble sugars and sugar-phosphates, and quantification of starch.

Table S4. Flux modelling data.

erac187_suppl_Supplementary_Figures_S1-S4

erac187_suppl_Supplementary_Tables_S1-S4

## Data Availability

The proteomics data are available in the EMBL-EBI Protein Identification Database PRIDE with the project accession number PXD029901.

## Abbreviations

ACO: 1-aminocyclopropane-1-carboxylic acid oxidase
DBE: starch debranching enzyme
DPE: disproportionating enzyme
F6P: fructose 6-phosphate
G1P: glucose 1-phosphate
G6P: glucose 6-phosphate
GLP: germin-like protein
PHS: glucan phosphorylase
PWD: phosphoglucan water dikinase
SuSy: sucrose synthase
WAE: weeks after bunch emergence
