# Supplementary material for: From fruit growth to ripening in plantain: a careful balance between carbohydrate synthesis and breakdown
Source: J Exp Bot. 2022 May 5;73(14):4832–49. doi: 10.1093/jxb/erac187 (PMC9366326; doi:10.1093/jxb/erac187)
Supplement: erac187_suppl_Supplementary_Figures_S1-S4 [file erac187_suppl_supplementary_figures_s1-s4.pdf]

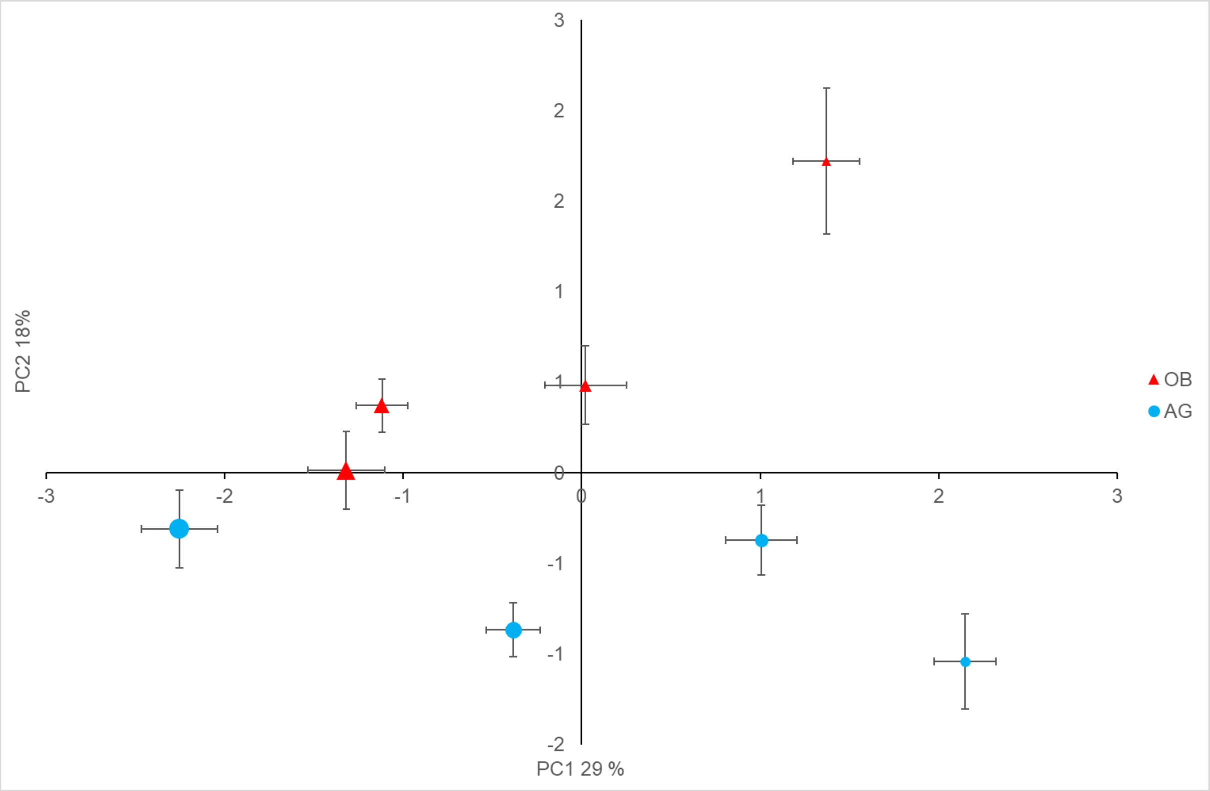

FIGURE S1

A:Principal Component Analysis of the metabolic compounds (amino acids, F6P, fructose, G1P, G6P, glucose, malate, polyphenols, starch, sucrose, protein content, cell wall) of the two varieties of plantain banana during fruit development. Scores plot. AG: Agbaba ; OB: Obino l'Ewai. The size of the data points is proportional to the time of sampling. Pulp samples were analyzed at 6, 8, 10 and 12 WAE, n =3-5. Error bars indicate standard error.

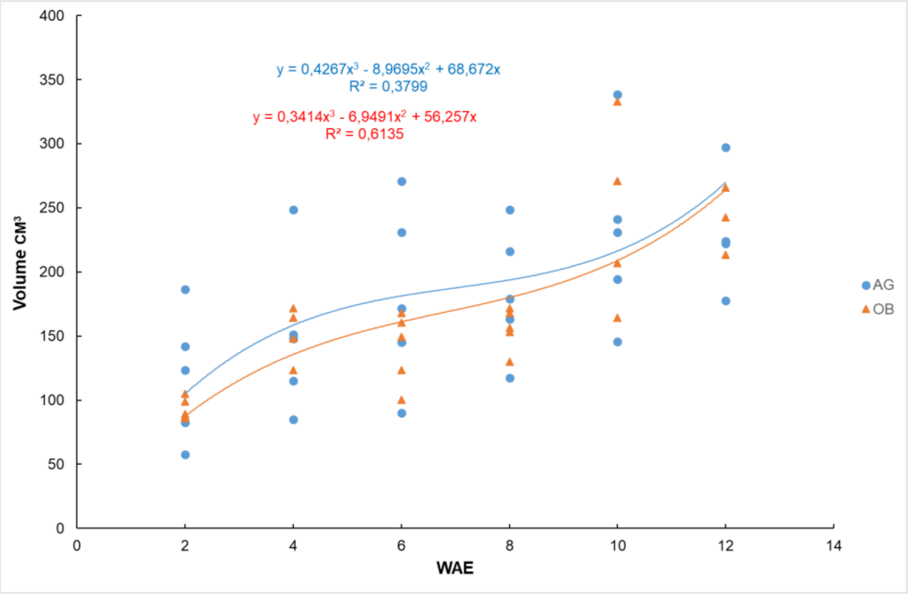

Figure S2: **Fruit volumes in function of time for the two plantain varieties.** **Samples have been harvested at 2, 4, 6, 8, 10 and 12 Weeks After bunch Emergence. n =3-5. AG: Agbaba ; OB: Obino l'Ewai.**

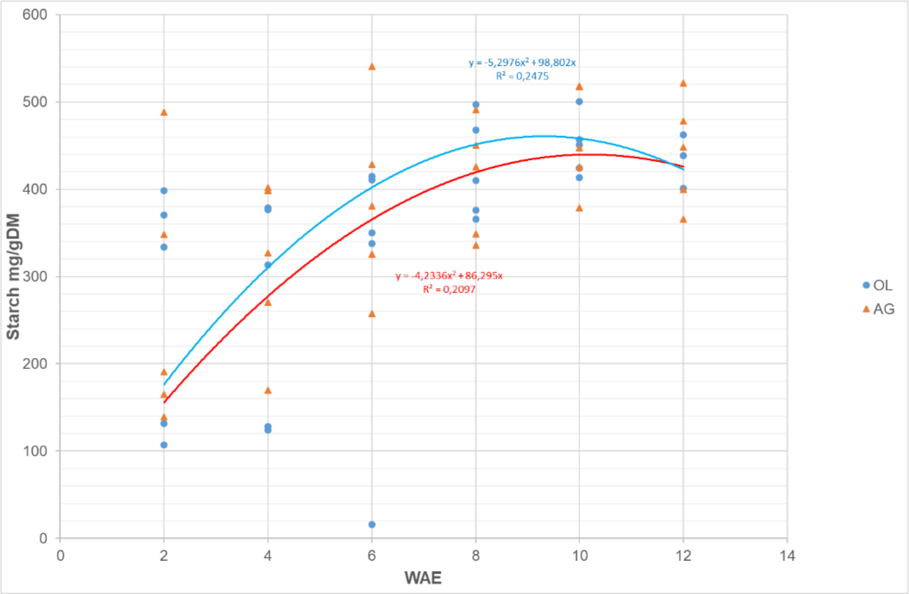

Figure S3: Starch contents in function of time for the two plantain varieties Samples have been harvested at 2, 4, 6, 8, 10 and 12 WAE. n =3-5. AG: Agbaba ; OB: Obino l'Ewai.

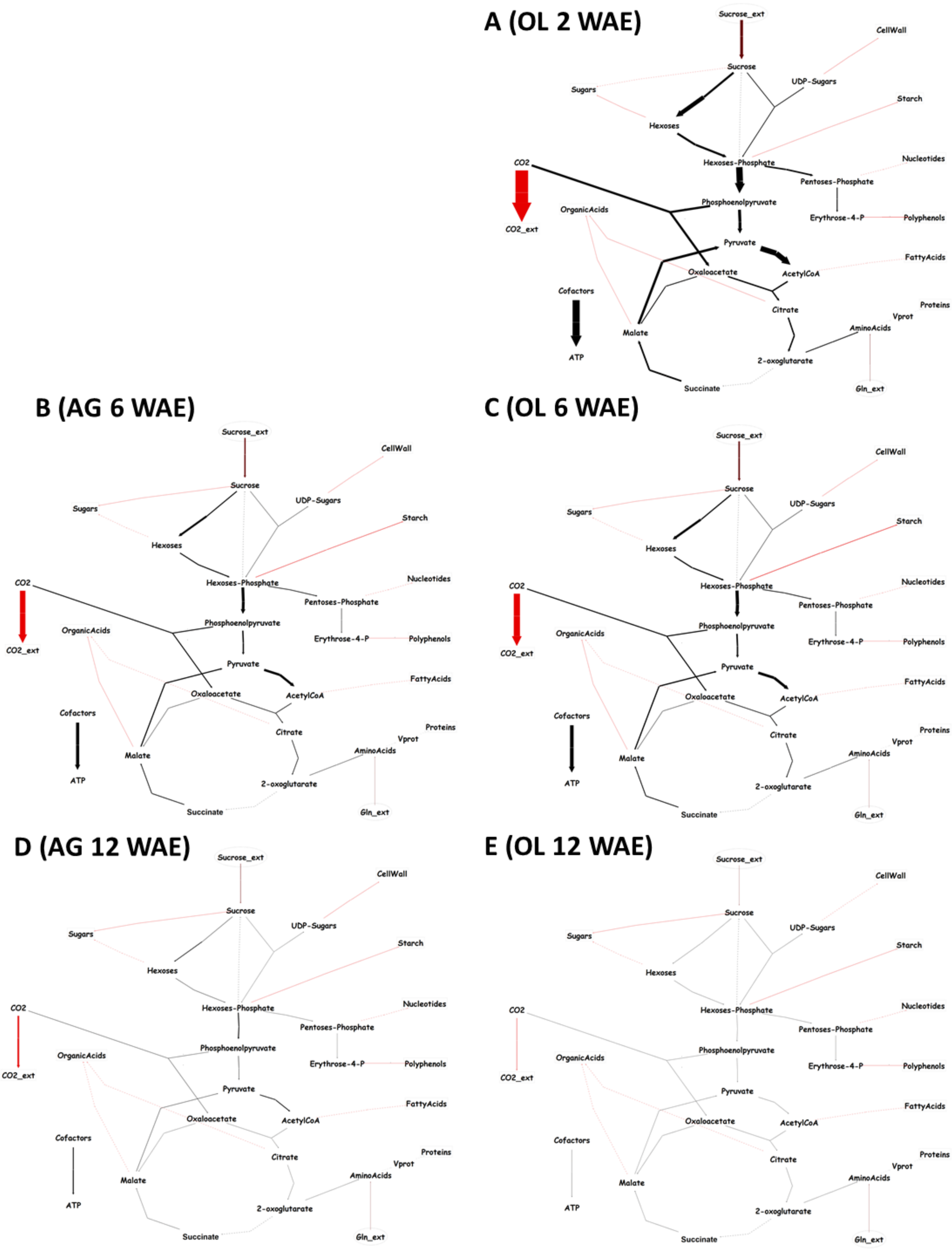

A (OL 2 WAE)
B (AG 6 WAE)
C (OL 6 WAE)
D (AG 12 WAE)
E (OL 12 WAE)
Sucrose_ext
Sucrose
CellWall
UDP-Sugars
Sugars
Starch
Hexoses
Hexoses-Phosphate
Nucleotides
Pentoses-Phosphate
Erythrose-4-P
Polyphenols
CO2
CO2_ext
Phosphoenolpyruvate
OrganicAcids
Pyruvate
AcetylCoA
FattyAcids
Oxaloacetate
Citrate
Cofactors
ATP
Malate
2-oxoglutarate
AminoAcids
Vprot
Proteins
Succinate
Gln_ext

Figure S4: **Simplified flux map, based on constraint-based modelling for OB (A, C and E) and AG (B and D) at 2 WAE (A), 6 WAE (B and D) and 12 WAE (D and E), showing a high activity for fluxes in glycolysis, TCA cycle and mostly respiration (in red). The arrow width is proportional to flux intensity. Maps were drawn with the flux visualization tool of VANTED 2.1.0**.
